# Self-rated health and objective health status as predictors of all-cause mortality among older people: a prospective study with a 5-, 10-, and 27-year follow-up

**DOI:** 10.1186/s12877-020-01516-9

**Published:** 2020-03-30

**Authors:** Maarit Wuorela, Sirkku Lavonius, Marika Salminen, Tero Vahlberg, Matti Viitanen, Laura Viikari

**Affiliations:** 1 Faculty of Medicine, Department of Geriatrics, University of Turku, Turku City Hospital, Kunnallissairaalantie 20, FI-20700 Turku, Finland; 2 City of Turku, Welfare Division, Turku, Finland; 3 Joint Authority for Päijät-Häme Health and Social Care, Elderly Care and Rehabilitation, Salpausselkä Rehabilitation Hospital, Lahti, Finland; 4 Faculty of Medicine, Unit of Family Medicine, University of Turku, Turku, Finland; 5 Institute of Clinical Medicine, Biostatistics, University of Turku, Turku, Finland; 6 Division of clinical geriatrics, NVS, Karolinska Institutet and Department of Geriatrics Karolinska University Hospital, Huddinge, Stockholm, Sweden

**Keywords:** Frailty, Mortality, Objective health, Older people, Self-rated health

## Abstract

**Background:**

Despite a non-specific nature of self-rated health (SRH), it seems to be a strong predictor of mortality. The aim of this study is to assess the association of SRH and objective health status (OH) with all-cause mortality in 70-year-old community-dwelling older people in Finland.

**Methods:**

A prospective study with 5-, 10- and 27-year follow-ups. SRH (*n* = 1008) was assessed with a single question and OH (*n* = 962) by the Rockwood’s Frailty Index (FI). To assess the association of SRH and OH with mortality, Cox regression model was used.

**Results:**

Of the 1008 participants, 138 (13.7%), 319 (31.6%), and 932 deceased (86.3%) during the 5-, 10- and 27-year follow-ups, respectively. In unadjusted models, subjects with poor SRH had almost eightfold risk for mortality compared to those with good SRH during the 5-year follow-up; among those with poor OH, the risk was fourfold compared to those with good OH. In the 10-year-follow up, both poor SRH and poor OH predicted about fourfold risk for mortality compared to those with good health. During the 27-year follow-up, OH was a stronger predictor of mortality than SRH. Poor SRH, compared to good SRH, showed 95% sensitivity and 34% specificity for 5-year mortality; corresponding figures for OH were 54 and 80%, respectively.

**Conclusions:**

Single-item SRH seems to be able to capture almost the same as OH in predicting a short-term (less than 10 years) mortality risk among older adults in clinical settings. The use of SHR may also enhance the focus on patient-centered care.

## Background

Self-rated health (SRH) is a subjective indicator of health status that integrates biological, mental, social and functional aspects of a person, including individual and cultural beliefs and health behaviors [1]. Despite seemingly non-specific nature of SRH, it has shown to be an unusually strong predictor of mortality [2, 3]. The predictive value of SRH, in part, reflects its lack of definition. A lot of objective information is included in the self-assessment of health, probably even more than is usually possible to include in a survey instrument or even to gather in a busy clinical settings [4]. Nevertheless, single-item self-reported scales, such as SRH, is often overlooked by clinicians because they assume that it is less reliable and more sensitive to contextual effects than objectively assessed health status [5–7].

In the earlier meta-analysis of 22 studies, the duration of follow-ups ranged from 15 months to 25.1 year. The association between general SRH and mortality was similar for studies with greater than and for those with less than 5 years maximum follow-up. In 10 studies with follow-ups from 15 months to 12 years, participants were 65 years or older. However, no subgroups analyses by age were conducted [2]. Among older people, long-term predictive value of SHR has found to be poorer than that of short-term [8–11].

In this study, our main interest was to clarify whether a single-item SRH is valuable tool for risk assessment of older adults in busy clinical settings. Therefore, we analysed the association of SRH and objective health status (OH) with all-cause mortality in 70-year-old community-dwelling older people in Finland during the 5-, 10- and 27-year follow-up.

## Methods

### Study participants

The study population of this prospective follow-up study consisted of all 70-year-old community-dwelling older adults of the city of Turku (The Turku Elderly Study) in Southwestern Finland, who were born in 1920. Data were collected in 1990–1991 by using postal questionnaires, interviews and clinical examinations. The protocol of data collection and flow chart are described in detail previously [12]. One thousand thirty-two (83%) participants completed the whole study protocol, but those with missing data of SRH (*n* = 24) were excluded, leaving 1008 participants for this study. They were followed up for 27 years for all-cause mortality.

### Data collection

SRH was measured by using a single question “in general, how would you rate your health” with reply alternatives: 1. good, 2. rather good, 3. poor, and 4. very poor. Responses “poor” and “very poor” were combined because of low number of responses in category “very poor” (*n* = 31), creating a three-category SRH variable: 1. good, 2. rather good, and 3. poor.

Rockwood’s comprehensive and multidimensional FI, which characterizes the whole health of individual [13], was used as a measure of objective health status (OH). FI consists of at least 30 deficits, such as symptoms, signs, disabilities, diagnoses, and laboratory measurements, which are readily available in survey or clinical data [14, 15]. In this study, FI consisted of 36 items described in Additional file 1. For any individual, FI was calculated as the number of deficits divided by the number of items considered [15]. In this study, OH was classified according to the level of FI [16, 17] as follows: 1. good (FI ≤0.08, robust), 2. moderate (FI 0.09–0.24, pre-frail), and 3. poor (FI ≥0.25, frail) [18].

Data of potential confounding factors were collected by using postal questionnaires and interviews. Sociodemographic variables used were age, gender, living circumstances (1. living with someone, 2. living alone), and education (1. ≤8 years, 2. ≥9 years). Need for help was assessed with three variables (yes/no): need for daily help, having help from a relative at least once a week, and having someone who helps when needed.

Cognitive function was measured by Mini-Mental State Examination [19]. Cognitive impairment was defined as a score lower than 27 [20, 21]. Charlson comorbidity index (1. 0–1, 2. ≥2) [22, 23] was used to record presence of chronic comorbid disease.

Psychosocial status was measured with questions concerning feelings of depression (1. never or seldom, 2. sometimes, often or always), loneliness (1. never or seldom, 2. sometimes, often or always), and life satisfaction (1. yes, 2. no).

Weight and height were measured, and body mass index (BMI), measured as kilograms per square meter, was calculated. Weight change during the past 3 years was assessed as follows: 1. increased, 2. no change, and 3. decreased. Health behavior was measured with questions concerning smoking (1. never, 2. stopper or smoking) and having daily outdoor activities (1. yes, 2. no). Physical functioning was assessed with difficulties in walking 500 m (1. no, 2. yes) and number of falls during the previous 12 months (1. no falls, 2. 1–2 falls, 3. ≥3 falls).

The study Data from all participants who died before January 2017 were obtained from the official Finnish Cause of Death Register using unique personal identification numbers.

### Statistical analyses

Baseline differences by SRH were analyzed using the Chi-squared test and Fisher’s exact test. Hazard ratios (HRs) and their 95% confidence intervals (CI) for all-cause mortality were calculated using Cox proportional hazard models. Proportional hazards assumption was tested by using Martingale residuals. The follow-up periods were calculated from baseline measurements to the end of the follow-ups of 5-, 10- and 27-year or to the death of the person.

Firstly, unadjusted Cox regression analyses were conducted for SRH and OH. Secondly, Cox regression analyses for OH was adjusted for gender and SRH for those possible confounding factors which were significantly associated with higher mortality risk at the follow-ups of 5-, 10- and 27- year and which did not have high between-correlation. The interaction between genders was included in Cox regression models. Thirdly, interaction between OH and SRH was analyzed, genders together and separately, in order to examine whether SRH gives any extra value in evaluation of mortality risk by OH.

*P*-values less than 0.05 were considered statistically significant. All statistical analyses were performed by using SAS System for Windows, version 9.4 (SAS Institute Inc., Cary, NC, USA).

## Results

### Baseline characteristics

Overall, 10.2, 63.9, 25.9% of the participants reported having good, rather good, or poor SRH, respectively. Table 1 shows the baseline characteristics of the participants by SRH. Ninety percent of the participants with good SRH also had good OH; participants with poor SRH, 7, 49, and 44% were categorized as having good, moderate, or poor OH, respectively (*p* < 0.001) (Table 2). SRH was highly correlated with OH was (Spearman’s correlation coefficient 0.57, *p* < 0.001).

**Table 1:** Baseline characteristics of the community-dwelling 70-year-old people (*n* = 1008) by self-rated health

	Good (*n* = 103) n (%)	Rather good (*n* = 644) *n* (%)	Poor (*n* = 261) *n* (%)	*P*-value
Women	67 (65)	437 (68)	142 (54)	<.001
Living alone	39 (38)	283 (44)	97 (37)	.127
Education≥9 years	37 (38)	114 (18)	34 (13)	<.001
Need for daily help	0 (0)	15 (2)	61 (24)	<.001
Help from a relative at least once a week	19 (19)	142 (23)	111 (43)	<.001
Having someone who helps when needed	86 (85)	509 (81)	211 (81)	
Mini-Mental State Examination < 27	26 (25)	217 (34)	104 (40)	.018
Charlson’s comorbity index ≥2	57 (58)	373 (60)	175 (71)	.005
Feelings of depression at least sometimes	16 (16)	178 (28)	131 (51)	<.001
Feelings of loneliness at least sometimes	6 (6)	152 (24)	102 (40)	<.001
Satisfaction with life	98 (98)	613 (97)	224 (88)	<.001
Body mass index, kg/m^2^				.257
< 25	43 (48)	189 (37)	57 (35)	
25–29.9	34 (38)	241 (47)	73 (45)	
≥ 30	13 (14)	86 (17)	32 (20)	
Weight change during the past 3 years				<.001
Increased	18 (18)	1616 (25)	75 (29)	
No change	72 (71)	374 (59)	122 (47)	
Decreased	12 (12)	96 (15)	60 (23)	
Smoking				.041
Has never smoked	60 (58)	381 (60)	133 (51)	
Has stopped or is still smoking	43 (42)	256 (40)	126 (49)	
Having daily outdoor activities	100 (97)	624 (98)	221 (86)	<.001
Difficulties in walking 500 m	5 (5)	71 (11)	116 (45)	<.001
Three or more falls during the previous 12 months	3 (3)	18 (3)	27 (10)	<.001

**Table 2:** Cross-classification of self-rated health by objective health status among community-dwelling 70-year-old people (*n* = 943)

	Self-rated health			*P*-value
	Good (*n* = 97) *n* (%)	Rather good (*n* = 604) *n* (%)	Poor (*n* = 242) *n* (%)	
Objective health status				<.001
Good	87 (90)	344 (57)	17 (7)	
Moderate	10 (10)	226 (37)	118 (49)	
Poor	0 (0)	34 (6)	107 (44)	

### Self-rated health and objective health status and risk of mortality

**Fig. 1:**
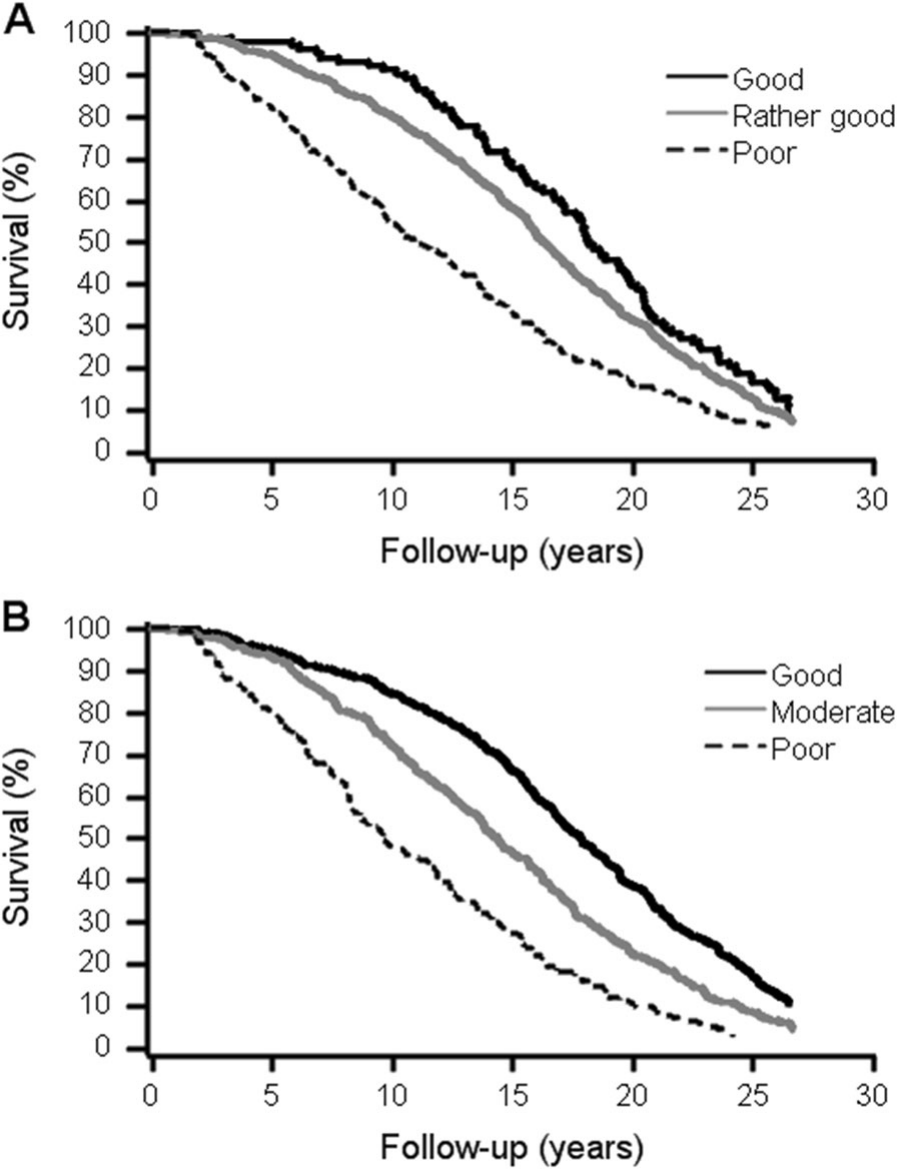
Kaplan-Meier survival curves by self-rated health (**a**) and objective health status (**b**). Number of participants alive at 0-, 5-, 10-, 15-, 20- and 25-year follow-ups by. **a** Self-rated health categories. Good: 451, 429, 381, 299, 175 and 76. Moderate: 363, 338, 262, 170, 81 and 30. Poor: 148, 119, 71, 40, 15 and 4. **b** Objective health status categories. Good: 103, 101, 94, 70, 41 and 17. Rather good: 644, 611, 515, 376, 203 and 81. Poor: 261, 213, 141, 86, 41 and 17

Of the total of 1008 participants, 138 (13.7%), 319 (31.6%), and 932 (86.3%) deceased during the 5-, 10- and 27-year follow-ups, respectively. According to both unadjusted and adjusted Cox proportional hazard models, poor SRH and both moderate and poor OH were associated with significantly higher mortality risk during the 5-, 10- and 27-year follow-ups (Tables 3 and 4). According to clinically more interesting unadjusted models, poor SRH was a stronger predictor of short-term (5- and 10-year follow-up periods) mortality than OH. Figure 1 shows the Kaplan-Meier curves of unadjusted analyses of SRH and OH for all-cause mortality during the total of 27-year follow-up. However, after adjustment of analyses of SRH with multiple confounding factors significantly associated with mortality, gender-adjusted OH slightly stronger predicted mortality than SRH in 5-, 10-, and 27-year follow-up.

**Table 3:** Unadjusted hazard ratios (HR) and their 95% confidence intervals (CI) (in parentheses) of self-rated health and objective health status for all-cause mortality among home-dwelling 70-year-old people during the 5-, 10- and 27-year follow-up

		5-year follow-up		10-year follow-up		27-year follow-up	
Self-rated health (*n* = 1008)	*n* (%)	HR (95% CI)	*P*-value	HR (95% CI)	*P*-value	HR (95% CI)	*P*-value
Good	103 (10)	1		1		1	
Rather good	644 (64)	2.60 (0.95–7.13)	.064	1.79 (1.07–2.99)	.026	1.23 (0.99–1.53)	.067
Poor	261 (26)	7.89 (2.88–21.61)	<.001	4.59 (2.73–7.70)	<.001	2.01 (1.58–2.56)	<.001
Objective health status (*n* = 962)							
Good	451 (47)	1		1		1	
Moderate	363 (38)	1.60 (1.05–2.45)	<.001	1.93 (1.48–2.52)	<.001	1.50 (1.30–1.74)	<.001
Poor	148 (15)	3.98 (2.57–6.16)	.030	3.85 (2.86–5.18)	<.001	2.57 (2.12–3.12)	<.001

**Table 4:** Adjusted hazard ratios (HR) and their 95% confidence intervals (CI) (in parentheses) of self-rated health and objective health status for all-cause mortality among home-dwelling 70-year-old people during the 5-, 10- and 27-year follow-up

		5-year follow-up		10-year follow-up		27-year follow-up	
Self-rated health (*n* = 1008)	*n* (%)	HR (95% CI)	*P*-value	HR (95% CI)	*P*-value	HR (95% CI)	*P*-value
Good	103 (10)	1^a^		1		1	
Rather good	644 (64)			2.29 (1.24–4.23)^c^	.009	1.19 (0.94–1.51)^d^	.143
Poor	261 (26)	2.17 (1.42–3.31)^†^	<.001^b^	4.08 (2.14–7.77)^‡^	<.001	1.62 (1.23–2.13)^d^	<.001
Objective health status (*n* = 962)							
Good	451 (47)	1		1		1	
Moderate	363 (38)	1.62 (1.06–2.48)^e^	.027	1.99 (1.53–2.60)^e^	<.001	1.62 (1.40–1.87)^e^	<.001
Poor	148 (15)	4.13 (2.67–6.40)^e^	<.001	4.20 (3.12–5.66)^e^	<.001	2.91 (2.40–3.54)^e^	<.001

^a^Categories “rather good” and “good” are combined for statistical analyses because of the lack of deceased among subjects with a good self-rated health

^b^Adjusted for variables associated with 5-year mortality: gender, education, need for daily help, having someone (relative) who helps when needed, smoking, weight change, ability to walk 500 m, comorbidity, and life satisfaction, (*n* = 882)

^c^Adjusted for variables associated with 10-year mortality: gender, education, need for daily help, having someone (relative) who helps when needed, smoking, weight change, ability to walk 500 m, feelings of depression, and life satisfaction, (*n* = 910)

^d^Adjusted for variables associated with 27-year mortality: gender, need for daily help, having someone (relative) who helps when needed, smoking, weight change, ability to walk 500 m, falls, feeling of depression, and life satisfaction, (*n* = 908)

^e^Adjusted for gender

The association of SRH and mortality did not significantly differ between men and women at any given follow-up; both moderate (*p* = 0.047) and poor OH (*p* = 0.015) predicted significantly higher mortality risk among men (HR 1.85 [95% CI 1.46–2.34] for moderate OH; 3.59 [2.59–4.97] for poor OH) than women (1.45 [1.21–1.73]; 2.51 [1.97–3.21], respectively) during the 27-year follow-up.

We also analyzed interaction between OH and SRH in order to examine whether SRH gives any extra value in evaluation of mortality risk by OH. Analyses were conducted genders together and separately. No statistically significant interactions were found at any given follow-up.

### Sensitivity and specificity of self-rated health and objective health status for 5-year mortality

We also analyzed sensitivity and specificity of poor SRH and poor OH for 5-year mortality. When poor SRH was compared to good SRH, sensitivity and specificity were 95 and 34%, respectively, whereas in comparison of poor SRH with rather good or good SRH, sensitivity and specificity were 51 and 78%, respectively. Poor OH compared to good OH showed 53% sensitivity and 80% specificity. Corresponding figures for poor OH compared to moderate or good OH were 33 and 87%, respectively.

## Discussion

This study aimed to analyze the association of SRH and OH with all-cause mortality, showed that SRH performs well in comparison to OH. According to our results, in (clinically interesting) unadjusted models, poor SRH predicted mortality stronger than having a moderate or poor OH in 5- and 10-year follow-ups. After adjustments of SRH with multiple variables, which were significantly associated with higher mortality risk at given follow-ups, the association of poor SRH with mortality was still almost as strong as the association of poor OH with mortality in 10-year follow-up. In 27-year follow-up, although the predictive value of OH was higher than that of SRH poor self-rated health still predicted higher mortality risk. In consistence with our results, several earlier studies have shown that poor SRH is associated with higher mortality risk [2, 6, 24–26] but long-term predictive value of SHR has found to be poorer than that of short-term among older adults [8–11]. Anyhow, it seems that the subjective feeling of one’s health may be able to capture the same if not more than comprehensive, multidimensional and time-consuming objective assessment of health for prediction of mortality risk [27]. According to Jylhä [3], good SRH is no guarantee of true health, but poor SRH certainly needs further attention. However, SRH did not give any extra value in evaluation of mortality risk by OH at any given follow-up.

Single-item self-reported scales, such as SRH, are often overlooked as they are perceived as less reliable and more sensitive to contextual effects [5–7]. However, SRH is a generally reliable measure, and at the population level, sufficiently stable [28]. In clinical settings, SRH can also serve as a screening tool for patients’ health status and to enhance patient-centered care by allowing the patient to express a point of view genuinely his or her own [3]. This supports the use of SRH as a simple measure both in survey and clinical settings to identify vulnerable older adults [26, 28]. There is also evidence that the validity of SRH is increasing. This is probably because individuals are including more objective information in their self-assessment of health. It is also possible that individuals are considering more mortality-related conditions than they did in the past. Therefore, SRH should not be overlooked when it comes to understanding individuals’ health [24].

The findings of previous studies have been contradictory in relation to gender differences in the association of SRH and mortality [10]. In our study, the predictive value of SRH did not significantly differ between men and women at any given follow-up. Having moderate or poor OH, instead, predicted significantly higher mortality risk among men than women in 27-year follow-up. In our study, comprehensive and multidimensional FI, which characterizes the whole health of individual, we used as a measure of OH. According to the results of a meta-analysis, women had higher FI scores than men but lower mortality rate at any given level of frailty or age. This suggests that women tolerate frailty better than men [29].

The strengths of this study were a large sample size with a high response rate and a long follow-up period. SRH was assessed by a global SHR question, which has found to be more accurate for prediction of mortality in old age than for example, comparative question [30]. In our study, no time-dependent covariates [9] were used; proportional hazard assumption of baseline SRH and OH were tested by using Martingale residuals. Analyses for SRH were also adjusted for multiple confounding factors that were significantly associated with higher mortality risk at every given follow-up. It is possible, that this has led to over-adjustment, since factors, such as gender, education, need for daily help, ability to walk 500 m, feelings of depression and life satisfaction, may mediate the association between SRH and mortality.

## Conclusions

Findings of our study provide additional evidence supporting the value of incorporating a single-item measure of SRH into risk assessment of older adults in busy clinical settings. Routinely collecting SRH may also enhance the focus on patient-centered care.

## Supplementary information

**Additional file 1.** Variables used in the assessment of objective health status

## Data Availability

The datasets used and/or analysed during the current study are available from the corresponding author on reasonable request.

## Abbreviations

BMI: Body mass index
CI: Confidence intervals
FI: Frailty index
HR: Hazard ratio
OH: Objective health status
SRH: Self-rated health
