# Supplementary material for: Self-rated health and objective health status as predictors of all-cause mortality among older people: a prospective study with a 5-, 10-, and 27-year follow-up
Source: BMC Geriatr. 2020 Mar 30;20:120. doi: 10.1186/s12877-020-01516-9 (PMC7106830; doi:10.1186/s12877-020-01516-9)
Supplement: Supplementary file 1 — Additional file 1. Variables used in the assessment of objective health status [file 12877_2020_1516_MOESM1_ESM.docx]

Appendix 1. Variables used in the assessment of objective health status

Has long-term disability or handicaps

Restriction of activities

Needs help for preparing meals

Needs help for shopping for necessities

Needs help for house work

Needs help for heavy household chores

Needs help for personal care

Needs help for moving about inside house

Has arthritis or rheumatism

Has high blood pressure

Has chronic bronchitis or emphysema

Has diabetes mellitus

Has heart disease

Has cancer

Has stomach or intestinal ulcers

Suffers from the effect of stroke

Suffers from urinary incontinence

Hip or femoral fracture

Falls within 12 months

Cardiorespiratory capacity (NYHA)

Has other medical conditions

Have no regular exercise

Has vision problem

Has hearing problem

Feeling hopeless

Has dexterity problem

Has emotional problem

Has memory problem

Has bodily pain

Has speech problem

Taking 5 or more medications

Has difficulty carrying or lifting light loads

Mobility problem

Has limited kind or amount of activity

Feels tired all the time

Weight loss
